# Determining the feasibility and effectiveness of brief online mindfulness training for rural medical students: a pilot study

**DOI:** 10.1186/s12909-020-02015-6

**Published:** 2020-04-06

**Authors:** Sarah Moore, Rita Barbour, Hanh Ngo, Craig Sinclair, Richard Chambers, Kirsten Auret, Craig Hassed, Denese Playford

**Affiliations:** 1 Rural Clinical School of WA, University of Western Australia, Busselton, Australia; 2 Rural Clinical School of WA, University of Western Australia, Perth, Australia; 3 Centre of Excellence in Population Ageing Research, University of New South Wales, Sydney, Australia; 4 Monash University, Melbourne, Australia; 5 Rural Clinical School of WA, University of Western Australia, Albany, Australia; 6 Department of General Practice, Monash University, Melbourne, Australia

**Keywords:** Online mindfulness training, Stress management, Rural medical education, Self-compassion

## Abstract

**Background:**

We sought to determine the feasibility and effectiveness of a mindfulness training program, delivered online to medical students at a Rural Clinical School.

**Methods:**

An 8-week online training program was delivered to penultimate-year medical students at an Australian Rural Clinical School during 2016. Using a mixed methods approach, we measured the frequency and duration of participants’ mindfulness meditation practice, and assessed changes in their perceived stress, self-compassion and compassion levels, as well as personal and professional attitudes and behaviours.

**Results:**

Forty-seven participants were recruited to the study. 50% of participants were practising mindfulness meditation at least weekly by the end of the 8-week program, and 32% reported practising at least weekly 4 months following completion of the intervention. There was a statistically significant reduction in participants’ perceived stress levels and a significant increase in self-compassion at 4-month follow-up. Participants reported insights about the personal and professional impact of mindfulness meditation training as well as barriers to practice.

**Conclusions:**

The results provide preliminary evidence that online training in mindfulness meditation can be associated with reduced stress and increased self-compassion in rural medical students. More rigorous research is required to establish concrete measures of feasibility of a mindfulness meditation program.

## Background

Medical students experience high levels of stress during their training [1–4] and this continues in their junior doctor years [5, 6], potentially resulting in depression, anxiety [7], burnout [8], alcohol abuse [9], and suicidal thinking [10]. Stress and poor mental health increase the risk of unprofessional behaviour including impaired decision-making [11], increased errors [12, 13], reduced attention and concentration [13], cynicism and loss of compassion [14] and impaired interpersonal skills [15]. Mindfulness training may be an important strategy for reducing stress and its negative consequences in medical students, and such programs are already delivered in some medical schools [16–26]. However, the number of controlled studies published is small [27].

Mindfulness involves paying attention on purpose, in the present moment and non-judgmentally [28]. It can be practiced formally, through meditation, and informally, by consciously bringing awareness to each moment of each day [28].

Mindfulness practice can lead to increased levels of self-compassion, regulating stress and optimising coping [29, 30]. Self-compassion consists of three main elements: self-kindness, a sense of common humanity, and a balanced, mindful relationship with unpleasant thoughts and emotions [30]. Mindfulness may also increase empathy and compassion for others, although to date, studies have mainly measured changes in empathy [24, 31] despite some discussion that compassion may be healthier than empathy for healthcare professionals [32]. While empathy involves sharing in the suffering of another, compassion is feeling warmth, concern and care along with the motivation to improve another’s wellbeing. Whereas empathy can lead to emotional contagion and ultimately vicarious trauma and burnout, compassion appears to avoid this by promoting self-other distinction more explicity [32]. This has been shown to lead to greater positivity, kindness and prosocial behaviour [32]. The Medical Board of Australia [33] highlights the value of self-care and compassion for others as important professional behaviours in their Code of Conduct [33].

Many effective mindfulness training interventions already trialled in medical schools require up to 2 h of face-to-face training per week and 30–45 min of practice per day, for 5 to 8 weeks [16–18, 22–24, 26]. This is a significant time commitment, which may be unfeasible given the content-load in medical school curricula. It has also been reported that medical students may not have the motivation to practice mindfulness meditation for long periods [34]. Research from Australia’s Monash University, which embedded mindfulness training into their core medical curriculum 25 years ago, has found that as little as 10 min of regular daily practice improves medical student wellbeing, study engagement and quality of life [34]. Other research suggests that it is the amount of formal meditation practice done between lessons, rather than lesson duration or program length, that predicts outcomes [35, 36].

Online delivery methods may also be an important strategy for increasing the accessibility and impact of mindfulness training for medical students. One pilot study explored an online intervention of three one-hour modules followed by self-reflection exercises for health professional trainees and practicing professionals [37], demonstrating a small but significant immediate increase in mindfulness scores. Online delivery may support the many medical educators who do not feel confident or qualified to teach mindfulness to their students [28] and provide equitable acess to students who learn in rural or distributed faculties.

The lead author of this pilot study SM, with support from authors CH and RC, developed an 8-week online mindfulness training programme (MTP) for rural medical students including brief, regular mindfulness practices. The content of the MTP was based on the teachings of authors CH and RH who have been teaching mindfulness to medical students for 20 years. The format was chosen based on the evidence suggesting online methods may be appropriate. The programme’s feasibility and effectiveness was assessed using self-report tools and reflective essays. SM, CH and RH all have expertise in mindfulness and medical education.

## Methods

### Design

This was a single-arm prospective mixed method cohort study. The design did not include a control group as a major focus was on feasibility. Feasibilty was defined as the proportion of participants who still practised formal mindfulness by the conclusion of MTP, and the frequency and duration of practice. The effectiveness of the MTP was determined by measuring changes in participants’ perceived stress level and reported compassion for self and others from baseline to 8-weeks, and whether such changes were sustained at 4-month follow-up. The statistical predictivity of the frequency and duration of mindfulness practice for the extent of the changes in the participants’ stress and compassion levels was also examined. Participants were invited to provide reflective essays that were qualitatively analysed, allowing deeper exploration of factors underpinning quantitative changes and whether these were associated with students’ self-reported professional behaviours.

### Participants

Students from an Australian Rural Clinical School were recruited in April 2016. They were penultimate-year students, who spent the entire academic year in one of 14 rural towns, with Australian Standard Geographical Classification – Remoteness Area scores of RA2-RA5 [38].

Recruitment involved emailing the 2016 cohort (*n* = 87) an invitation to attend a 45-min video-conferenced lecture, in which the first author described the project. A follow-up email then invited students to participate in the study. The email invitation was sent by the program facilitator, SM, who is also a senior lecturer at the Rural Clinical School. Participation was voluntary and required signed informed consent. Students were informed that the decision to participate would have no impact upon academic marks. No other formal mindfulness instruction was included in the Rural Clinical School curriculum during the year.

### Intervention

The MTP was delivered over 8 weeks and is summarised in Tables 1 and 2.

**Table 1:** MTP outline

Component	Duration	Delivery method	Frequency	Content
Mini-lecture	10 min	Emailed hyperlink to audiovisial recording	Weekly (every Monday)	1. Multi-tasking versus effective task switching
				2. Stress reduction and performance
				3. Reducing distraction and procrastination
				4. Mindful communication
				5. Regulating emotions
				6. Compassion
				7. Self-compassion
				8. Mindful use of technology
Guided mindfulness meditation session	5 min	SMS’ed. hyperlink to audio recording	Daily (Monday to Friday)	1. Body scan
				2. Mindful breathing
				3. Thought-labelling
				4. Mindful listening
				5. Working mindfully with emotions
				6. Loving-kindness
				7. Self-compassion
				8. Mountain meditation

**Table 2:** Educational Objectives of Mini-Lectures

Mini-lecture	Educational Objectives
Multi-tasking vs effective task switching	• Understand the negative consequences of multi-tasking, eg mistakes, fatigue, miscommunication
	• Learn how mindfulness practice promotes a positive alternative to multi-tasking: effective task-switching
Stress reduction and performance	• Understand the negative impact of excess stress on academic and clinical performance
	• Learn how to moderate stress using mindfulness to improve performance
Reducing distraction and procrastination	• Understand how distraction and procrastination results from lack of mindful awareness
	• Learn how to reduce these limiting behaviours using mindfulness practice
Mindful communication	• Understand how habitual and reactive communication styles can negatively impact on relationships with colleagues and patients
	• Learn how to practice mindful communication to optimize these relationships
Regulating emotions	• Understand the nature of emotions and how they impact on our behaviours and interactions with others
	• Learn how mindfulness practice allows us to notice and regulate our emotions and behaviour
Compassion	• Define compassion and explore why it is important for medical students to practice this skill
	• Understand how compassion can be developed through mindfulness practice
Self-Compassion	• Define self-compassion and why it is important for medical students to practice this skill
	• Understand how self-compassion can be developed through mindfulness practice
Mindful use of technology	• Understand how uncontrolled use of technology can negatively impact clinical performance and relationships
	• Learn mindfulness strategies for using technology efficiently

As a further support, the first author SM was available to participants via email or telephone, although none of the participants accessed this support individually. SM met with participants in small groups via videoconference between weeks 3–6 of the MTP to provide encouragement and answer questions.

### Tools

The Perceived Stress Scale (PSS) is a widely used 10-item self-report instrument, which measures the degree to which situations in an individual’s life are perceived as stressful [39]. Participants respond to each item using a scale ranging from 0 (never) to 4 (very often). Total possible scores range from 0 (minimum) to 40 (maximum); higher scores indicate higher levels of perceived stress. The Self-Compassion Scale (SCS) is a widely used 26-item self-report instrument that assesses components of self-compassion [40]. Responses are given on a scale ranging from 1 (almost never) to 5 (almost always). Total possible scores range from 26 to 130; higher scores indicate higher levels of self-compassion. The Compassion Scale (CS) is a 24-item self-report instrument to assess compassion for others. Responses are given on a scale ranging from 1 (almost never) to 5 (almost always). Total possible scores range from 24 to 120; higher scores indicate higher levels of compassion for others [41].

The PSS, SCS and CS were completed at baseline, at conclusion of the MTP and at 4-month followup. Other data collected via email survey at baseline included demographics (age, gender, ethnicity and home university), details of any prior training in mindfulness, and whether participants already had a regular mindfulness practice. During the intervention, a weekly survey was administered via email to record the frequency and duration of mindfulness practice during the previous week (Additional file 1). Participants were also asked to report the frequency and duration of any mindfulness practice they had undertaken between completion of the MPT and 4-month follow-up. Within 4 weeks of the end of the program, participants were asked to submit a 500-word reflective essay, exploring how the MTP had influenced their personal and professional attitudes, beliefs and behaviours (Additional file 2).

### Quantitative analysis

Data on key outcome measures were first summarized descriptively. The statistical significance of stress and compassion score changes were then assessed using dependent-sample t-test; and statistical predictors of score changes were assessed using generalized linear modelling (GLM). Candidate statistical predictors considered in the GLM models incuded the participants’ age, gender, ethnicity, prior exposure to mindfulness, and current practice of mindfulness. Data were collated in the REDCap application, and exported to Excel for cleaning and then SAS (version 9.4) for statistical analyses. Statistical significance was set at α = 0.05.

### Qualitative analysis

Qualitative data from the reflective essays was subjected to thematic analysis [42]. The first two authors SM and RB read through each essay and identified codes, which were organized into categories, then a thematic framework developed. Co-authors CS and RC applied this thematic framework on an independent analysis of a subset of essays. Themes most relevant to the research questions on feasibility and effectiveness of the intervention were agreed upon by the team.

## Results

### Sample description

Forty-seven medical students (54% of the year cohort) were recruited into the study via email following a mid-semester introductory lecture about mindfulness. Their baseline characteristics are described in Table 3. Of these, 34 (72%) provided data at week 8 and 28 (60%) at 4 months. Thirteen participants (27%) who completed the MTP submitted a reflective essay. Students who completed the 8-week program and provided their weekly practice data (‘completers’) did not statistically differ from ‘non-completers’ with respect to baseline characteristics.

**Table 3:**
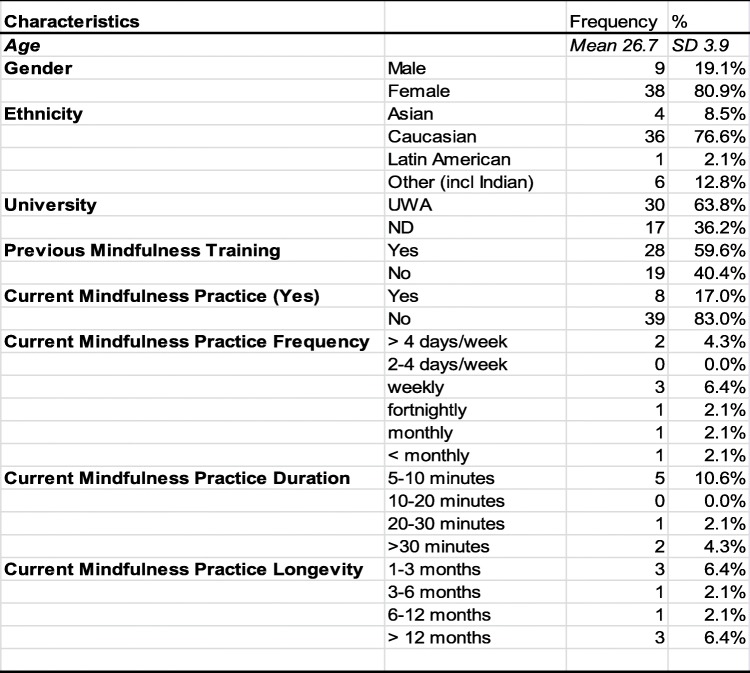
Characteristics of participating students at baseline (*N* = 47)

### Frequency and duration of mindfulness practice

**Fig. 1:**
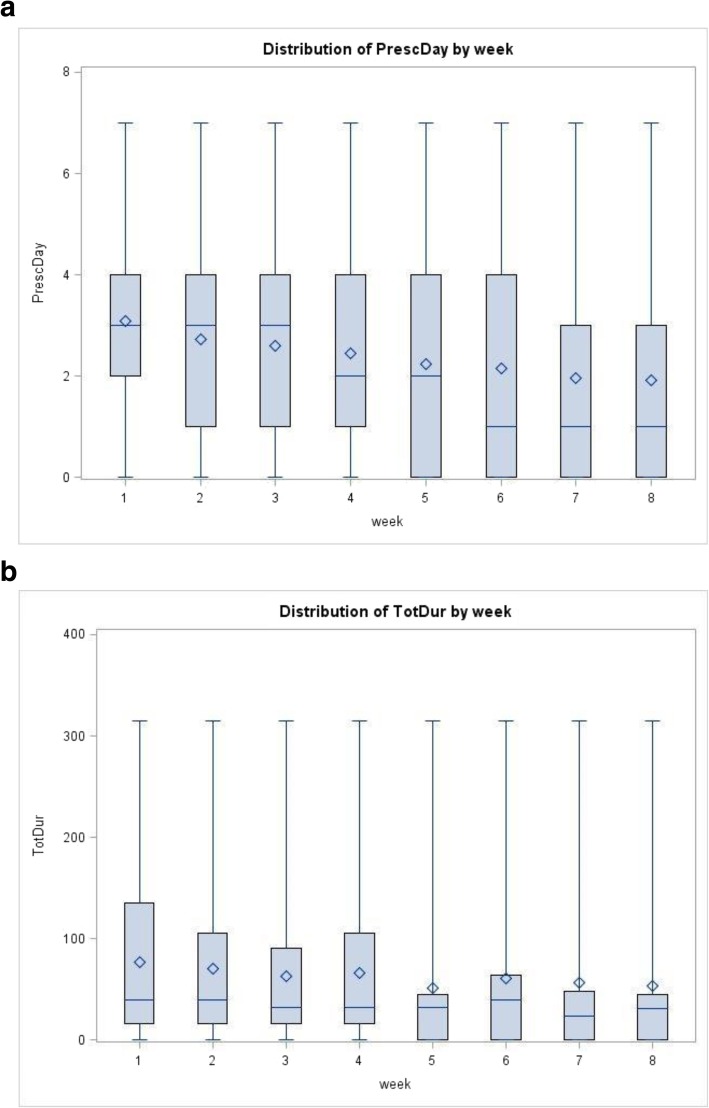
**a** Boxplots of weekly prescribed mindfulness meditation practice – Total number of days. **b** Boxplots of weekly mindfulness meditation practice – Total duration estimated in minutes. Note: As seen in Additional file 2, original data collected on duration of mindfulness practice were in ‘units of time’ not necessarily of equal intervals. To give these ‘time-units’ a more practical meaning, we approximated (or replaced) them with their mid-point values in minutes (i.e., 8 minutes for category 2 (between 5 and 10); 15 minutes for category 3 (10-20 minutes); and 25 minutes for category 4 (20-30 minutes). Category 1 (0-5 minutes) was ascribed with a value of 5 minutes if the participant had indicated that they practiced MM on that particular day, as we inferred that the students would have practiced MM for 5 minutes as per the prescribed exercise, rather than for 3 minutes (i.e., the mid-point value). Category 5 (>30 minutes) was conservatively ascribed with 45-minute duration

The boxplots in Fig. 1a show that at least half the participants practiced mindfulness a minimum of 3 days a week in the first 3 weeks, but practice then dropped over the following 5 weeks. Fig. 1b shows that the median and mean duration of total mindfulness practice remained relatively stable over the weeks; by Week 8, about half of the students were practicing for approximately 30 min in total per week. At 4-month follow-up, 11 (32%) participants continued to practice at least weekly. Most (*n* = 25, 89%) practiced for 10 min or less per session.

### Changes in PSS, SCS, and CS scores

Table 4 shows the participants’ PSS scores were significantly reduced at 4-month follow-up, compared to baseline (estimate − 2 points, CI -3.9, − 0.1). Participants’ SCS scores also increased significantly at both 8 weeks (estimate + 6.3, CI 1.2, 11.5) and 4 months (estimate + 5.5 points, CI 1.0, 9.9). Changes in CS scores were not significant.

**Table 4:**
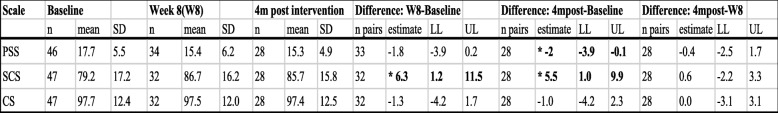
Scores on Perceived Stress Scale (PSS), Self Compassion Scale (SCS), and Compassion Scale (CS), before (i.e., at baseline) and after MTP

*LL* Lower Limit of 95% Confidence Interval. *UL* Upper Limit of 95% Confidence Interval

* denotes statistically significant effects at alpha = 0.05

Note: In this study’s context, stress (as measured by the PSS) is an undesired construct; as such, a negative score change (i.e., stress reduction) was desired. Conversely, compassion for self and others (as measured by the SCS and CS, respectively) is desired; accordingly, a positive score change (i.e., increase in compassion) is desired

### Predictors of PSS, SCS, and CS score changes

Frequency and duration of mindfulness practice did not predict changes in PSS, SCS or CS. There was a strong baseline effect for stress and self-compassion score changes. Those reporting higher stress at baseline observed greater stress reduction at 4-month follow-up. Similarly, lower self-compassion at baseline was associated with greater improvement in self-compassion at 4 months (see Table 4).

There was a gender effect for PSS score change, with males reporting on average 4.8 points (CI 0.9. 8.8) less stress reduction than females. However, when baseline effect was concurrently considered, the gender effect disappeared (*p* = 0.2646), whereas the baseline effect persisted (*p* = 0.0055). Note that these results are not shown in Table 5.

**Table 5:**
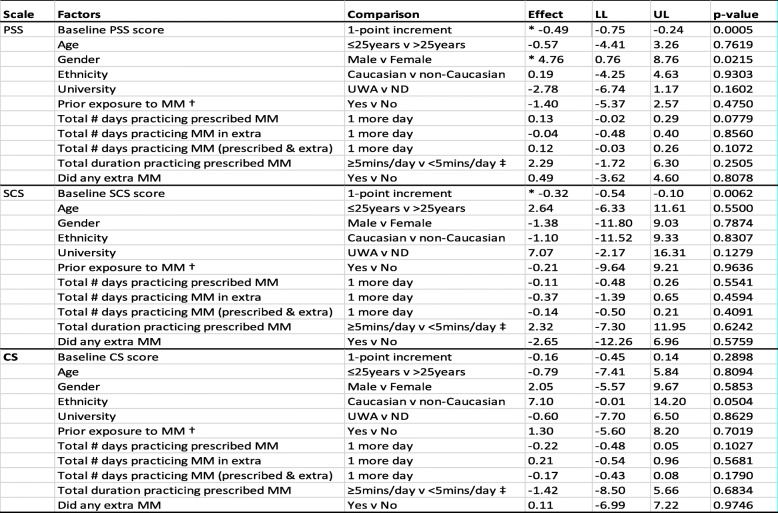
Statistical significance of potential factors contributing to changes in scores on PSS, SCS, and CS, between 4 months post-intervention and baseline

*MM* Mindfulness meditation

† includes previous training and/or existing practice in mindfulness meditation

‡ total duration over 8-week course (i.e., 56 days) is calculated at a minimum of 5 min × 56 days = 280 min. Models using median total duration (331 min) as cutoff threshold also yielded similar (non-significant) results

*LL* Lower Limit of 95% Confidence Interval. *UL* Upper Limit of 95% Confidence Interval

* denotes statistically significant effects (*p* < 0.05)

### Qualitative assessment of students’ reflections on the MPT

Two main themes were identified: how the participants engaged with the mindfulness program (including motivation for and barriers to engagement plus perception of content); and the impact of mindfulness practice on personal and professional attitudes, beliefs and behaviours.

#### Engagement

A number of the participants commented that they found the program “*beneficial*”, “*valuable*” and “*enjoyable*”. Some noted that they were motivated to learn techniques that would improve their performance and to develop coping strategies to manage stress and anxiety:

“*Prior to beginning the mindfulness program, I did not have a set of core coping strategies for accepting any anxious or distressing thoughts….Pracitising mindfulness meditations and watching the teachings has motivated me to continue to build my mindfulness skills*.” (P1)

It was challenging for some to remain engaged. Participants reported feeling overloaded by tasks and assessments, both in the medical curriculum and extra-curricular activities; difficulty prioritising mindfulness over other tasks; not enjoying some of the mindfulness practices; struggling to find a particular time of day to complete the practice; and not feeling like they need to practise every day especially when they were feeling “*happy*”. Despite these barriers, most went on to acknowledge the value of mindfulness and the benefits that they perceived even if their practice was irregular.

The content of the intervention appeared to influence participants’ interest and consequent practice. One mentioned that they “*related well to a couple of the mindfulness recordings*” which in turn raised their awareness of “*the discussed concepts in day to day life*” and “*extended to interactions with patients*” (P6). Another participant commented “*I really enjoyed the ‘body scan’, ‘awareness of breathing’ and ‘tuning into your surroundings’ meditations*” and found these easier to complete on a daily basis, however “*found the last four meditations very difficult… hence struggled to complete the meditation…and was not as compliant when it came to the second half of the program.*” (P2) This participant acknowledged “*I should have persisted and tried to improve, but instead chose not to complete the meditation some days because I found it too frustrating.*”

#### Impact

Many participants reported that practising mindfulness impacted on self-awareness of thoughts, emotions and behaviour. Participants described an increased awareness of the nature of the mind, including mind-wandering, the arising of emotions and habitual reactions to these emotions, self-criticism, stress and anxiety about doing things correctly, judgement of others, procrastination and constant thinking and analysis.

Mindfulness practice was described as providing opportunities to acknowledge thoughts and emotions non-judgementally and consequently chosing to respond in a more controlled way. For example, one participant said *“when I was more mindful, I felt more in control of myself”* (P1) whilst another explained *“I have been working towards pausing before reacting and understanding that emotions are transient, but the impact of our behaviour as a result of these emotions can have a lasting effect. This change in attitude and behaviour has allowed me to remain calm when in situations that are potentially volatile.”* (P5)

Some recognised that mindfulness practice was helpful in developing self-compassion:

*“The Mindfulness Program helped me look at my situation objectively and think about how I would judge someone else in the same circumstances. This made me more forgiving of how I was performing, which in turn made me aware of how tiring and self-defeating it was to be expending energy on self-criticism, when in fact I could be expending the same energy on looking forward and working towards achieving my goals.” (P11)*

Increased compassion for others was also reported by participants. One described how the loving kindness meditation helped “*to identify personal judgement towards patients*” and consciously “*maintain compassion towards them*”(P8). Another commented how their increased awareness of “*I’m too busy*” self-talk allowed them to make more time to help others.

Stress reduction was another outcome identified in the essays, as a consequence of becoming more aware of their anxious thoughts and emotions and having access to tools that allowed them to become present. One participant reported *“Through the mindfulness program, I have become more aware of acknowledging my thoughts in a state of non-judgemental awareness. I am now acknowledging my thoughts and choosing to redirect my focus. No longer do I feel annoyed with myself if these thoughts return, I simply acknowledge them and refocus” (P1).*

Most described using mindfulness reactively when they were under acute stress and feeling overwhelmed. For example, one participant reported “*in situations where I feel overwhelmed, for any reason, I have found that focussing on my breathing has been the most useful technique to helping me to return to a state of calm*” (P1). Another participant described their practice as *“my daily escape*” that “*took my mind off the daily stressors, even if just for 5 minutes*” (P7). Conversely, a small number reported that their daily mindfulness practice altered their experience of stress and allowed them to embrace life more fully. One participant stated “*Through becoming more aware of the emotions I am experiencing, I have been able to recognise how my body is responding to these emotions... In recognising when I am stressed or experiencing an emotion… I am able to stop myself, acknowledge that I’m feeling that way and accept the emotion without converting it to an unwanted action*” (P9). One participant commented that “*I was more inclined to practice mindfulness when I was relaxed. At times when I was stressed…I was too wound up to sit and relax*” (P2).

Some participants mentioned that practising mindfulness led to improved performance and productivity, as a consequence of increased self-awareness of thoughts and emotional regulation and new techniques for managing tasks and focussing their attention. One participant explained “*when I am in the hospital and I have deadlines…focusing on my breathing has helped me to calm my mind enough to break down my larger tasks into smaller ones. In this way, I am able to apply what was taught in the mindfulness videos and focus more on the process of completing my tasks rather than the outcome. I have found this to be extremely effective in increasing my productivity and decreasing my stress levels*” (P1).

## Discussion

The use of online mindfulness training with rurally-located medical students has not been documented previously in the medical education literature, and hence is a novel method which has shown to be feasible for medical students. Our study demonstrated an association between the program and reduction in perceived stress and increase in self-compassion levels in a cohort of rural medical students. It has also highlighted the need for ongoing innovation to create programs that are perceived as highly feasible and engaging for medical students, and for further research on ideal course duration and content.

### Mindfulness and stress reduction

There was no immediate post-training impact on perceived stress levels in this study. There are other pilot studies in health discipline trainees [16, 20] that similarly did not detect a significant change in PSS scores soon after completion of the mindfulness intervention, while one 4-week pilot program [19] did. Two RCTs [21, 25] have demonstrated statistically significant reduction in PSS at completion of five and eight-week mindfulness-based training programs, respectively. The difference in all these findings has yet to be adequately explained, but may relate to different content of the actual interventions, the timing over the academic year and other elements of the support provided by investigators that are not fully articulated.

The impact on perceived stress levels was seen at 4-month follow-up, interestingly just prior to the students’ final exams. This suggests that a time-limited mindfulness training progam may have a later effect on stress reduction. Warneke & Quinn [26] conducted follow-up of PSS at 2 months post-intervention, and likewise found that improvements in PSS were maintained. Although Moore [20] found no change in quantitative PSS scores following a 4-week mindfulness program, their qualitative data suggested a positive impact on meta-cognitive processes and a need for ongoing regular mindfulness practice for stress management. Our qualitative data indicate that mindfulness was primarily used by participants to distract themselves from stress, rather than actively managing it (i.e. by practising acceptance and learning to defuse from stressful thought patterns). Anecdotally, participants in mindfulness programs often use mindfulness in this way, although there is a paucity of research on this subject. Further research is warranted to examine exactly how participants use mindfulness to reduce stress, particularly given the well-established link between experiential avoidance and psychopathology [43].

### Mindfulness and compassion for self and others

Self-compassion levels increased by the end of the progam and remained elevated 4 months later. Two other studies [16, 25] found significant improvements in self-compassion scores following mindfulness intervention with students, consistent with our results, and strengthening the evidence that mindfulness training can have a significant and sustained effect on self-compassion. As highlighted by Mills and Chapman [44] being self-critical and perfectionistic is common amongst doctors, however being kind to oneself is important not only for preventing depression and burnout, but also for providing sustainable compassionate care to others. Curricula that specifically focuses on self-compassion, as done explicitly in week 7 of this MPT, may be especially beneficial, along with implicit training in self-compassion inherent in aspects of mindfulness such as acceptance and non-judgement of thoughts and emotions.

This study demonstrated no quantitative change in participants’ compassion levels at completion of the program or at follow-up. Despite this, some participants did report increased levels of compassion for others as a result of their mindfulness practice in their qualitiative essays. One explanation for the absence of change in compassion scores is that students had insufficient time or content to integrate the compassion teaching and meditations into their personal and professional lives before the completion of the program, and thus could not develop any further compassion over time. It is also possible that insufficient time was devoted to this topic, particularly because the delivery of the compassion component was in week 6 of the 8-week MPT, and coincided with a busier time for the participants’ academic schedules. Further, Mills and Chapman [44] note compassion may require initial development of self-compassion before compassion can manifest. Consequently it may be appropriate to first focus on developing mindfulness and self-compassion in future MPT interventions before graduating to compassion training.

### Feasibility of the MPT

The median frequency of mindfulness practice by week 8 was once per week. As a comparison, Monash University [34] has a well-established and longer duration programme, with 90.5% of students reporting practising mindfulness meditation at least once a week and also practicing informal mindfulness in their daily life. It is likely that the regular face-to-face contact between participants and facilitators during the Monash program led to a higher percentage of participants practicing weekly than in our online study. Hence it seems it is feasible to aim for a brief online MPT intervention to result in weekly mindfulness practice successfully incorporated into medical students’ busy schedules.

The qualitative data analysis adds valuable insight into potential motivators and barriers to engagement with mindfulness training and its impact on medical students’ personal and professional attitudes and behaviours. Some struggled to prioritize their mindfulness practice due to time pressures or content they did not like. However, confirming previous qualitative evaluations of mindfulness training programs, participants were able to write about valuable positive impacts including increased awareness and presence [18, 20, 45], more compassion to self and others [18, 45, 46], and improved emotional regulation and stress management [16–18, 20, 45–47] when they integrated the practice alongside other demands.

This intervention was purposively designed to have brief mini-lectures and guided meditations in a time-limited course as a means of making it feasible, however further work will be required to clarify if there is a minimum threshold for engagement to deliver meaningful outcomes.

Our study extends the current literature, demonstrating that positive and ongoing changes can occur with brief, weekly mindfulness teaching and meditation practice that is delivered online to rural medical students over an 8-week period.

### Strengths and limitations

This study had two major strengths, apart from the innovative method of online delivery. First, the duration of follow-up has not been achieved by other studies of this kind. Second, our mixed method provided a more comprehensive understanding of the underlying factors contributing to the changes in participants’ PSS and SCS scores.

There were a number of weaknesses in this study that may limit the applicability of our findings in other settings. Our design was a pilot study without a control group and comprised a self-selected sample. The small sample size limited the statistical power of the study. Further to this, there were low response and completion rates, which may be a source of bias. Participants had relatively low PSS and high SCS scores at baseline, suggesting a possible floor/ceiling effect. Each of these limitations could be addressed in future research using a randomised control trial design. The program facilitator was the principal investigator, which may have made it more likely for participants to self-report improvements, mindfulness practice and positive effects in their reflective essays. This was partially controlled by involving colleagues in coding the reflective essays. Delivering this program in other medical schools where the participants do not know the program facilitator may reduce this potential bias. The weekly survey used asked only about formal meditation practice, and assessment of students’ informal (i.e. mindfulness of everyday activities) was assessed only via qualitative essays and anecdotal reports. Future research should endeavour to include clear quantitative measures of both meditation and informal practice to adequately assess the relative conbtributions of each [34].

### Implications for future practice and research

Medical students with high PSS scores and low SCS scores at baseline benefited the most from the mindfulness training. This appears to be the first time this finding has been demonstrated. Future mindfulness training programs could therefore be targeted particularly towards individuals with high perceived stress and low self-compassion levels. The findings also suggest that a MTP that includes an explicit self-compassion focus may be an important strategy for helping medical students to develop their self-compassion and self-care. A randomised control trial in a larger cohort of medical students is now being designed to determine, with greater confidence, the feasibility and effeciveness of this MTP. Multi-trait, multi-method assessment is the most sophisticated and accurate research design [48] so future research using physiologic measures of stress, such as cortisol, could supplement self-reported measures to validate the effectiveness of stress-management techniques such as mindfulness training [49].

## Conclusion

This pilot study demonstrated the feasibility and effectiveness of an online 8-week mindfulness training program for reducing perceived stress, increasing self-compassion and compassion levels in rurally-located medical students. The intervention was associated with reduced perceived stress levels and increased self-compassion levels, and gave insights into developments which may improve participant engagement in future studies.

## Supplementary information

**Additional file 1.** Weekly survey.

**Additional file 2.** Qualitative Essay Question.

## Data Availability

The datasets analysed during the current study are available from the corresponding author on reasonable request.

## Abbreviations

MTP: Mindfulness Training Programme
PSS: Perceived Stress Scale
SCS: Self-Compassion Scale
CS: Compassion Scale
GLM: Generalized Linear Modelling
