# Supplementary material for: Determining the feasibility and effectiveness of brief online mindfulness training for rural medical students: a pilot study
Source: BMC Med Educ. 2020 Apr 6;20:104. doi: 10.1186/s12909-020-02015-6 (PMC7137339; doi:10.1186/s12909-020-02015-6)
Supplement: Supplementary file 1 — Additional file 1. Weekly survey. [file 12909_2020_2015_MOESM1_ESM.docx]

**Appendix I. Weekly survey**

During the past week, on which days did you complete your prescribed mindfulness meditation practice? (please highlight)

Monday Tuesday Wednesday Thursday Friday Saturday Sunday

Did you complete any extra mindfulness meditation practice this week? Yes No

If yes, what days? (please highlight)

Monday Tuesday Wednesday Thursday Friday Saturday Sunday

What was the **total** duration of your practice? (please tick)

|  | 0-5 mins | 5-10 mins | 10-20 mins | 20-30mins | >30mins |
| --- | --- | --- | --- | --- | --- |
| Monday |  |  |  |  |  |
| Tuesday |  |  |  |  |  |
| Wednesday |  |  |  |  |  |
| Thursday |  |  |  |  |  |
| Friday |  |  |  |  |  |
| Saturday |  |  |  |  |  |
| Sunday |  |  |  |  |  |
