# Supplementary material for: Determining the feasibility and effectiveness of brief online mindfulness training for rural medical students: a pilot study
Source: BMC Med Educ. 2020 Apr 6;20:104. doi: 10.1186/s12909-020-02015-6 (PMC7137339; doi:10.1186/s12909-020-02015-6)
Supplement: Supplementary file 2 — Additional file 2. Qualitative Essay Question. [file 12909_2020_2015_MOESM2_ESM.docx]

**Appendix II. Qualitative Essay Question**

“*We are interested to hear about your experience of the RCSWA Mindfulness Program. In particular, we would like you to comment on HOW the knowledge gained from the teaching videos and mindfulness meditation skills practiced with the audio recordings has influenced your personal and professional attitudes, beliefs and behaviours. Please limit your assignment to approximately 500 words.”*
